# Wide-angle metamaterial absorber with highly insensitive absorption for TE and TM modes

**DOI:** 10.1038/s41598-020-70519-8

**Published:** 2020-08-12

**Authors:** Majid Amiri, Farzad Tofigh, Negin Shariati, Justin Lipman, Mehran Abolhasan

**Affiliations:** School of Electrical and Data Engineering, University of Technology Sydney, Sydney, NSW 2007 Australia

**Keywords:** Electrical and electronic engineering, Metamaterials

## Abstract

Being incident and polarization angle insensitive are crucial characteristics of metamaterial perfect absorbers due to the variety of incident signals. In the case of incident angles insensitivity, facing transverse electric (TE) and transverse magnetic (TM) waves affect the absorption ratio significantly. In this scientific report, a crescent shape resonator has been introduced that provides over 99% absorption ratio for all polarization angles, as well as 70% and 93% efficiencies for different incident angles up to $\theta =80^{\circ }$ for TE and TM polarized waves, respectively. Moreover, the insensitivity for TE and TM modes can be adjusted due to the semi-symmetric structure. By adjusting the structure parameters, the absorption ratio for TE and TM waves at $\theta =80^{\circ }$ has been increased to 83% and 97%, respectively. This structure has been designed to operate at 5 GHz spectrum to absorb undesired signals generated due to the growing adoption of Wi-Fi networks. Finally, the proposed absorber has been fabricated in a $20 \times 20$ array structure on FR-4 substrate. Strong correlation between measurement and simulation results validates the design procedure.

## Introduction

Veslago investigated double negative (DNG) materials in 1968, since then new approaches have been found in the microwave regime^1^. DNG materials are well-known as metamaterials (MTMs) due to their superior characteristics. MTMs are 2D or 3D medium with a specific structure. 3D MTMs can be a lactic or regular 3D structure that are built using several components or printed by 3D printers. However, 2D MTMs are a layout of symmetric or non-symmetric resonators on dielectric substrate using printed circuit board (PCB) technology. MTM structures include numbers of unit cells that work as molecules in natural material. The electric and magnetic responses of MTMs are different compared to natural materials. Unlike natural materials, permittivity and permeability of MTM structures are negative. These properties lead to changing the behavior of electromagnetic (EM) waves facing MTMs. For instance, the Doppler effect and Snell’s law are no longer valid in the traditional way and work in opposite directions. The unusual characteristics of MTMs allow for interesting applications in antenna^2–4^, invisible cloak^5–8^, sensors^9–11^, superlens^12,13^, and many others.

Landy^14^, in 2008, introduced a new function for MTMs. He proposed the perfect absorber by utilizing the loss characteristic of the MTM substrate. Light weight, low profile and easy fabrication compared to traditional absorbers caused the rapid development of Metamaterial Perfect Absorber (MPA). Different applications have been exploited for MPA in various frequency ranges from microwave to terahertz. Absorption of undesired frequency^15–17^, THz applications^18–21^, thermal emitters^22,23^, optical switches^24–27^, sensors^28,29^ and energy harvesters^30–35^ are some of the applications made possible through the use of MPAs.

Various methods have been applied to tailor conventional metamaterial perfect absorber to be used in mentioned applications. For instance, the air gap has been added in the middle of the structure to keep the under-testing sample. Combination of dielectric substrate and added sample to MPA structure creates an effective medium that changes the absorption characteristics. This method has been mainly used in sensing applications^28,29^. In^36,37^, lumped resistors have been added to MPA structure in energy harvesting applications to avoid wasting trapped signals in lossy substrates and convert them to usable energy. It is noteworthy that adding lumped resistors increases absorption efficiency as well as bandwidth^16^. In^38^, four wheels resonator without lumped resistors shows multi absorption bands characteristics. After adding resistors, the adjacent absorption bands merge, and a broadband metamaterial absorber has been achieved.

Given the designs of the MPAs described above, insensitivity against different polarization and incident oblique angles is one of the main focal points in designing MPAs. Polarization insensitivity is a phenomenon that happens due to the symmetric shape of the unit cell vertically and horizontally^39^. However, considering the insensitivity facing TE and TM polarized waves makes incident angle insensitive structures more complicated. Different investigated unit cells such as circular sector^40,41^, multi-layers structure^42–44^, fractal structure^45,46^, surrounding via array^47^ and split ring resonator (SRR)^48,49^ mostly show good insensitivity for TM-polarized waves. Whereas, for TE-polarized waves, the absorption ratio falls dramatically by increasing the incident angle.

In this study, a semi-symmetric crescent shape resonator is introduced. The structure is investigated under normal incident waves to optimize its parameters with a full-wave simulation. The structure is fully polarization angle insensitive. Meanwhile, due to the field distribution on crescent-shaped blades, incident angle insensitivity can be adjusted for TE and TM polarized waves by changing structure parameters. The final structure is fabricated, and the experimental results are compared to the simulation results to confirm the design procedure.

## Unit cell design

**Figure 1:**
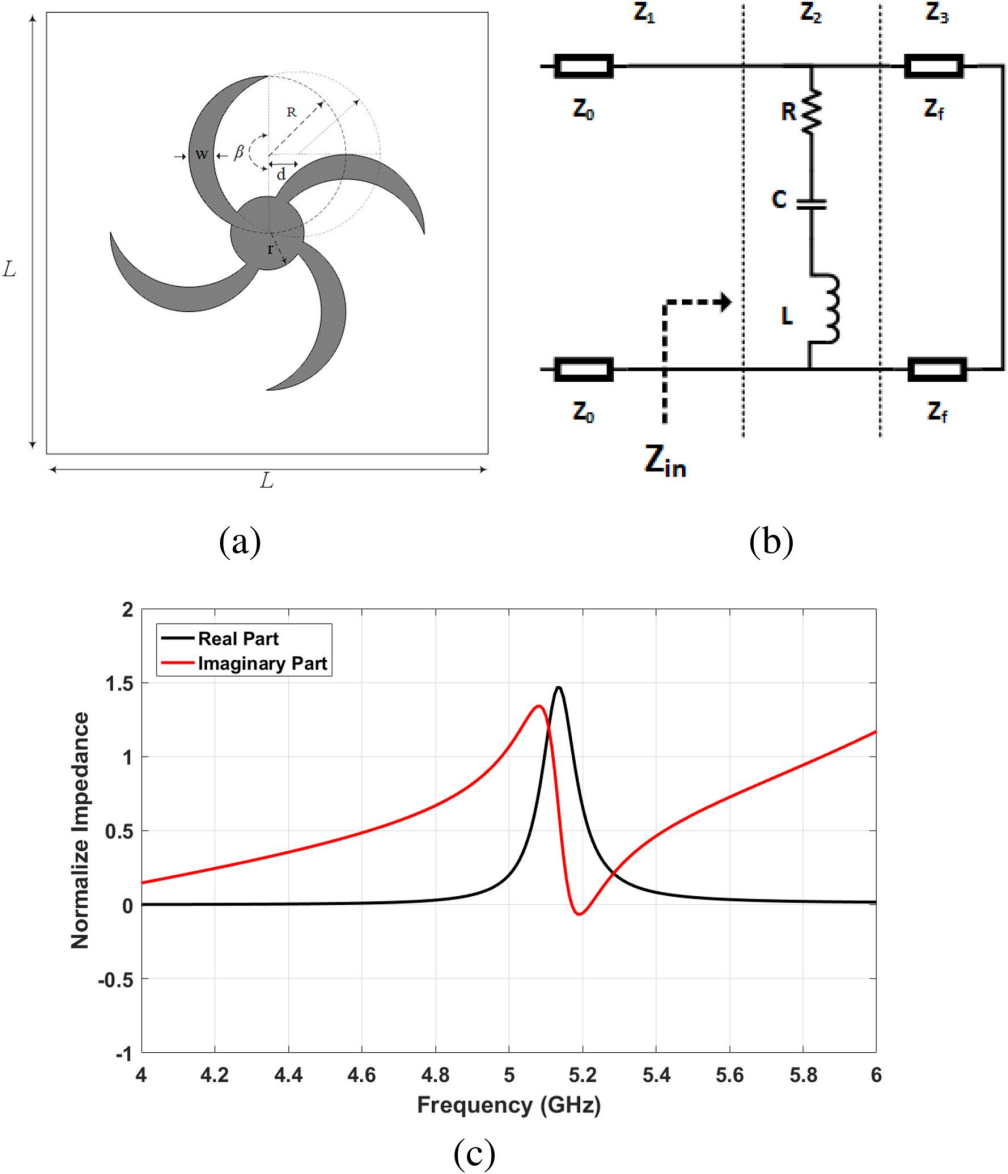
Proposed structure, (**a**) unit cell design, (**b**) equivalent circuit, (**c**) normalize impedance.

The final unit cell structure includes four crescent-shaped blades, as is shown in Fig. 1a. The crescent structure is created by subtracting a circular disk and a portion of another circle expelled from its edge. Hence, what remains is a shape enclosed by two round circular arcs of various diameters that converge at two points. The width and angle of the curve can be controlled by changing the radius and center position of the inner circle. In addition, the middle part is a circle that combines four blades.

**Figure 2:**
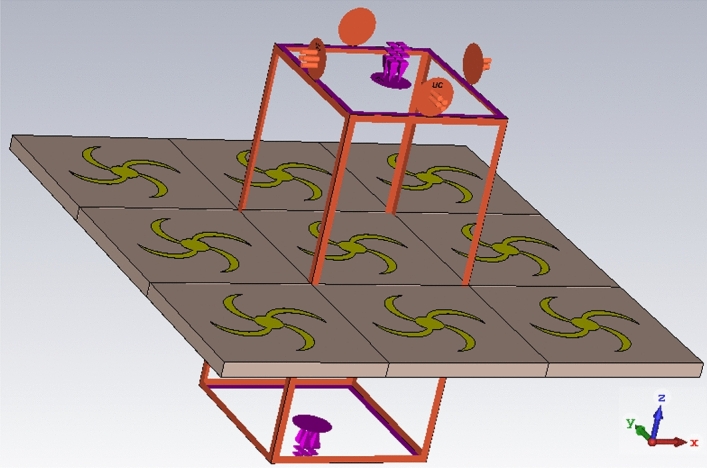
Numerical setup to analyse MPA structure.

To investigate the practicality of the proposed structure, CST Studio 2016 is used for simulation and numerical analysis. As is shown in Fig. 2, unit cell boundary condition is applied to the structure and Flouque port is used as an input excitation. This numerical setup allows for the analysis of the MPA under different polarization and incident angles.

Metamaterial absorbers are designed to be perfectly matched with intrinsic impedance of free space. The effective input impedance of MPA, $Z_{in}(\omega )$, is a function of the electric and magnetic responses, which leads to the effective electric permittivity $\varepsilon _{eff}(\omega )$ and magnetic permeability $\mu _{eff}(\omega )$. It should be noted that these parameters are functions of the frequency. Based on^50,51^, the effective input impedance of structure can be calculated according to Eq. (1):

1 $$\begin{aligned} Z_{in}(\omega )=\pm \sqrt{\frac{(1+S_{11}(\omega ))^2 -S_{21}^2(\omega )}{(1-S_{11}(\omega ))^2-S_{21}^2(\omega )}} \end{aligned}$$

**Figure 3:**
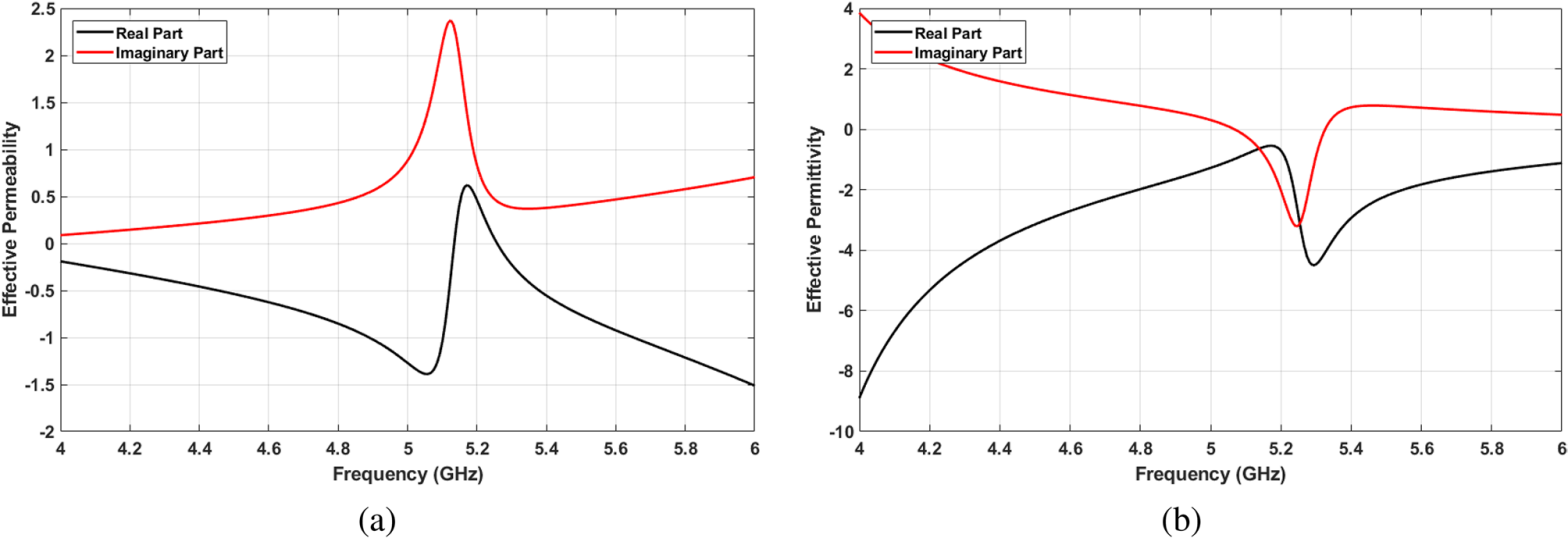
(**a**) Effective permeability, (**b**) effective permittivity.

The effective permittivity and permeability ($\varepsilon _{eff}$ and $\mu _{eff}$) are two main parameters to explain the metamaterial properties. These parameters can be obtained using the scattering parameter as well. However, the refractive index (n) of the structure has to be calculated based on Eq. (2) prior to determining permittivity and permeability:

2 $$\begin{aligned} n_{eff}(\omega )= \pm \left( \frac{1}{kL}\right) arccos\left( \frac{1-S_{11}^2+S_{21}^2}{2S_{21}}\right) \end{aligned}$$

where k and L denote the wavelength and the size of the unit cell, respectively. By calculating the MPA impedance and effective refractive index, effective permeability and permittivity are defined in Eqs. (3) and (4), respectively. Figure 3a,b show these two parameters. As can be seen in these figures, both effective permittivity and permeability have negative values around resonance frequency ($\varepsilon _{eff}<0$ and $\mu _{eff}<0$).

3 $$\begin{aligned} \mu _{eff}(\omega )= n_{eff}(\omega )Z_{eff}(\omega ) \end{aligned}$$

4 $$\begin{aligned} \varepsilon _{eff}(\omega )(\omega )= n_{eff}(\omega )/Z_{eff}(\omega ) \end{aligned}$$

To achieve perfect matching, the input impedance of structure should be equal to intrinsic air impedance ($Z_{in}(\omega )=Z_0$). Therefore, the reflection coefficient would be approximately zero ($\Gamma (\omega )=|S_{11} |^2 \approx 0$). Moreover, due to the metal plate on the back of the substrate, the EM wave transitivity is equal to zero ($T(\omega )=0$). As a result, the absorption of the structure is obtained according to Eq. 5:

5 $$\begin{aligned} A(\omega )=1-\Gamma (\omega )-T(\omega )=1- \left| \frac{Z_{in} (\omega )-Z_0}{Z_{in}(\omega )+Z_0}\right| ^2 \end{aligned}$$

The input impedance of the structure can also be computed using the equivalent circuit method. The transmission line model^52^ is used to analyse the equivalent circuit model, which is shown in Fig. 1b. Three sections are considered to extract the equivalent circuit:

- Section (1): The air with the intrinsic impedance of $$Z_0=377$$$$\Omega$$, which is shown by transmission line ($$Z_1$$).
- Section (2): The crescent shape resonator, which equalizes with RLC circuit ($Z_2$).
- Section (3): The dielectric substrate and the metal film on the bottom of structure that equalizes with a short circuit transmission line ($Z_3$).

6 $$\begin{aligned} Z_1= Z_0 \end{aligned}$$

7 $$\begin{aligned} Z_2(\omega )= R+j\omega L+ \frac{1}{j\omega C} \end{aligned}$$

8 $$\begin{aligned} Z_3(\omega )= jZ_f tan(\beta l)=j \sqrt{\frac{\mu _r \mu _0}{\varepsilon _r \varepsilon _0}} tan\left( \frac{2\pi }{\lambda } h\right) = 0.54277j \end{aligned}$$

9 $$\begin{aligned} Z_{in}(\omega )= Z_2(\omega )+Z_3(\omega ) \end{aligned}$$

where $$Z_f=179.71$$$$\Omega$$ is the impedance of FR-4 medium, $$\beta$$ is wave number, and l is the length of transmission line that is equal to dielectric height $$h=1.6$$ mm = $$\lambda /36.26$$ mm. Aiming to calculate values of R, L, and C, the input impedance has been extracted from $$Z_{in}(\omega)$$ chart. Figure 1c shows the normalized real and imaginary part of input impedance. The ideal impedance matching happens when the real and imaginary parts become 1 and 0, respectively. In this structure, the $$Z_{in}=1.053-j0.007$$ at $$f=5.17$$ GHz. Considering mentioned values, the $$R=0.854$$$$m \Omega$$, $$L= 124$$*pH* and $$C=6.5$$*pF* have been calculated.

## Numerical results

**Figure 4:**
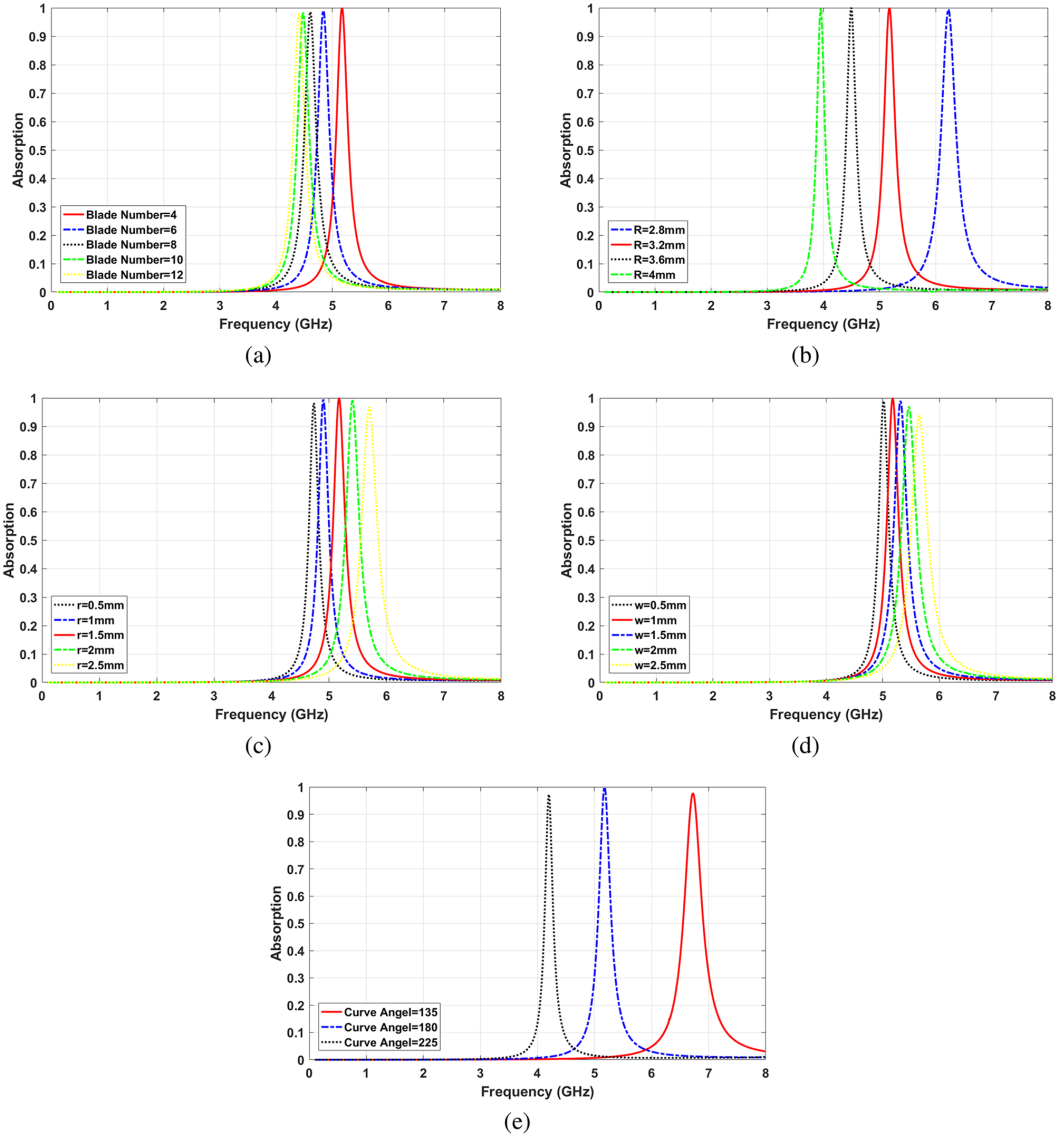
Effect of parameters (**a**) Number of blades, (**b**) crescent outer radius, (**c**) middle circle radius, (**d**) width of blades, (**e**) crescent curve angle.

The parametric study has been performed to investigate the effect of different geometrical parameters of the structure on absorption characteristic. The number of blades (*N*), width of each blade (*w*), radius of middle circle (*r*), radius of the outer circle (*R*)—which determines the resonance frequency—and the angle of the outer curve $(\beta )$ are investigated, and results are shown in Fig. 4.

Adding the number of blades (*N*) increases the metallic area of the resonator, and mutual effect between blades and the metal film, which are represented by increasing the values of the inductor and capacitor in the equivalent circuit, respectively. Consequently, the cut-off frequency of the structure shifts downward. However, the pace of moving the resonance frequency is decreased for the structures with more than ten blades, as it is shown in Fig. 4a. Moreover, it is seen that the absorption ratio is scaled-down continuously by adding the number of blades.

The radius of the outer circle (*R*) in crescent structure specifies the size of the blades and resonator. The resonance frequency shifts from 6.25 GHz for $R=2.8$ mm to 3.95 GHz for $R=4$ mm as shown in Fig. 4b. However, the absorption ratio does not change significantly by increasing *R*. The size of the middle circle (*r*) that connects the blades also shifts the frequency of the absorption band. Figure 4c illustrates that the absorption peak accrues in higher frequencies by increasing (*r*), while the highest absorption happens in $r=1.5$ mm. The increment of the absorption frequency is due to the reduction of the effective electrical length of the structure. In more detail, a larger unit area in the middle of the resonator leads to a shorter metal path. Consequently, the equivalent inductance of structure decreases notably, which causes lower cut-off frequency.

The distance between the center of two circles, considering their radiuses, determines the width of blades (*w*). As Fig. 4d shows, higher absorption frequency happens in case of wider blades. In addition to the described parameters, the crescent curve angle $(\beta )$ has a significant influence on resonance frequency. It can be used to shift the cut-off frequency downward and miniaturize the structure, as a result of extending the metallic path. In order to change $(\beta )$ from 135 to 225°, the absorption frequency shifts from 6.7 to 4.18 GHz, see Fig. 4e.

In this paper, the optimum values of parameters have been chosen to eradicate the 5 GHz band signals with the highest possible efficiency. Based on environmental measurement that performed at the University of Technology Sydney environment using a handheld spectrum analyzer, 5.17 GHz is the strongest frequency in 5 GHz spectrum in the tested area—this correlates with the lowest 5 GHz frequency channel used by WiFi routers. Hence, the peak point of the absorption band is set to be in this frequency. The final values are shown in Table 1.

**Table 1:** The optimum values of unit cell.

Parameter	L	N	R	r	w	$\beta$
Value	18 mm	4	3.2 mm	1.5 mm	1 mm	$180^{\circ }$

**Figure 5:**
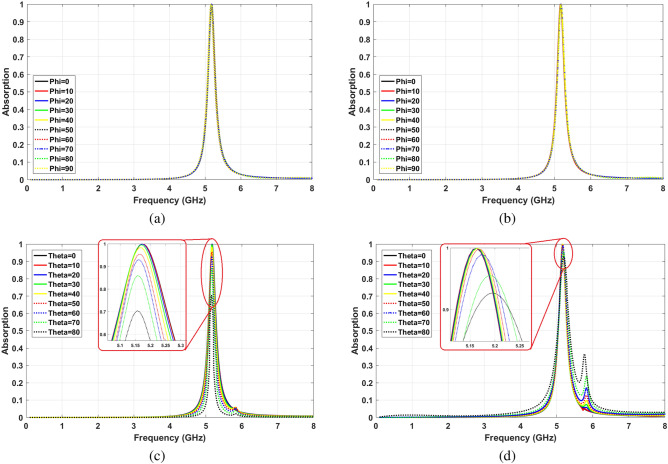
Angle insensitivity (**a**) different polarization angles for TE waves, (**b**) different polarization angles for TM waves, (**c**) different incident angles for TE waves, (**d**) different incident angles for TM waves.

Various electromagnetic waves with different polarization and incident angles exist in the environment. MPA has to be designed to absorb the maximum possible of radiated signals. The optimum values in Table 1 satisfy the highest level of absorption insensitivity for both TE and TM polarized waves. Figure 5 shows the absorptivity of proposed MPA for the different incident and polarization angles of waves. Based on the results of Fig. 5a,b, the final structure is perfectly polarization angle insensitive for both TE and TM modes waves. Moreover, Fig. 5c illustrates that the absorption frequency does not change with increasing incident angle up to $\theta =80^{\circ }$ for TE waves. Furthermore, the absorption ratio, which is higher than 95% at $\theta =50^{\circ }$ and below, is reasoably stable. It slightly decreases at $\theta =70^{\circ }$ and $\theta =80^{\circ }$ and becomes 86% and 70%, respectively.

For TM waves, as it is shown in Fig. 5d, shifting the absorption frequency is slightly more than TE waves; however, it is still less than 0.02 GHz. Furthermore, the absorptivity is highly insensitive against changing the incident angle. For instance, at $\theta =70^{\circ }$ and $\theta =80^{\circ }$ the absorption ratios are 96% and 92%, respectively.

**Figure 6:**
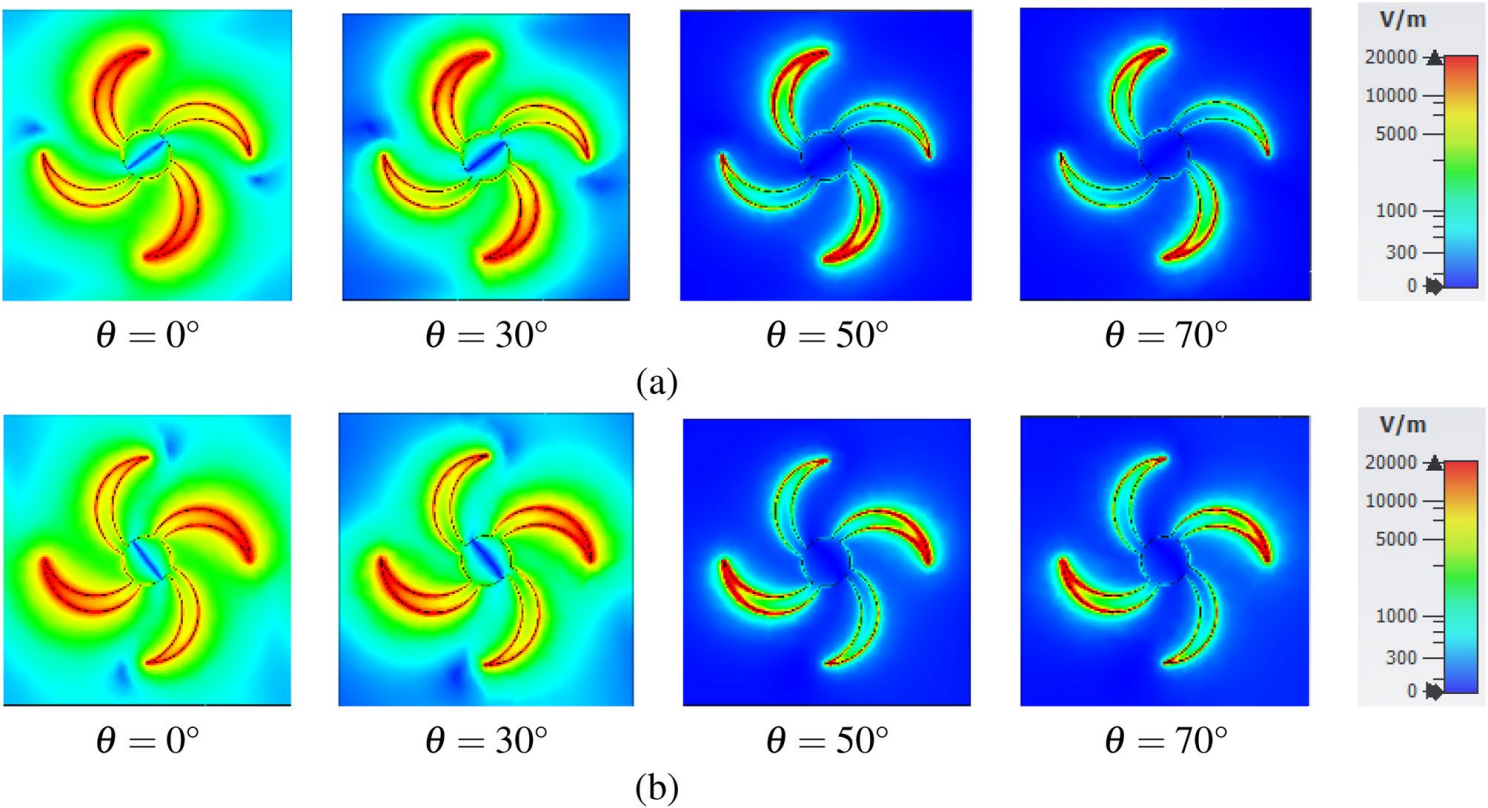
E-field distribution (**a**) TE polarized waves, (**b**) TM polarized waves.

Field distributions on the structure are provided in Fig. 6 to explain the mechanism of the absorption process facing TE and TM polarized waves. The E-field distributions confirm that the edges of blades have an essential role in the absorption process. Figure 6a,b illustrate the relatively stable attraction of electric field in these points for oblique incidence angles up to 70° for both TE and TM modes. The situation is almost the same for H-field distribution.

**Figure 7:**
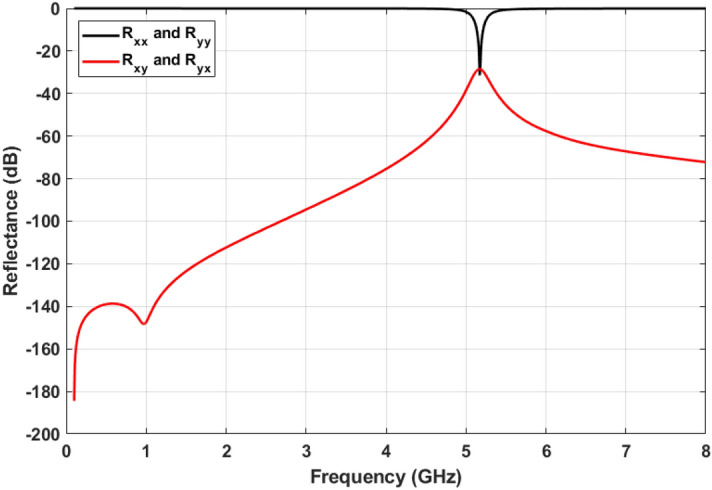
Co- and cross-polarization reflection.

As mentioned previously, the MPAs absorb the incident signal with near unity efficiency. However, it should be noted that this absorption efficiency has to be ideal for both co- and cross-polarization reflection. In the way of explanation, regardless of incident wave polarity, the reflected field from structure has components in both x and y directions. Considering these components, co-polarized reflection coefficients are defined as $R_{xx}=|E_{rx}|/|E_{tx}|$ and $R_{yy}=|E_{ry}|/|E_{ty}|$. In addition, cross-polarization reflection coefficient are determined as $R_{xy}=|E_{rx}|/|E_{ty}|$ and $R_{yx}=|E_{ry}|/|E_{tx}|$. Where $E_{tx}$ and $E_{ty}$ are define as the x-polarized and y-polarized components of transmitted waves, respectively. While, $E_{rx}$ and $E_{ry}$ are components of reflected waves. Applying the described boundary conditions in Fig. 2, $R_{xx}$ and $R_{xy}$ can be obtained, as shown in Fig. 7. Based on this figure both co- and cross-polarization reflection are almost − 30 dB which are negligible in absorption frequency.

In recent years, several polarization and incident angles insensitive MPA structures have been reported. Table 2 provides a comparison between designed metamaterial absorber and some of the reported structures. Several parameters such as polarization insensitivity, absorption ratio for TE and TM waves with different incident angles and shift of the absorption peak frequency for various angles have been investigated. In the designed structure, the absorption ratio is reasonably stable for incident angles up to $\theta =80^{\circ }$. In addition, the negligible shift in absorption peak frequency is another advantage of the designed structure.

**Table 2:** Comparison of proposed absorber performance with other insensitive absorber in literature.

Ref	Frequency (GHz)	Polarization insensitivity	$\theta =0^{\circ }$		$\theta =20^{\circ }$		$\theta =40^{\circ }$		$\theta =60^{\circ }$		$\theta =80^{\circ }$		Peak shift (GHz)	
			TE	TM	TE	TM	TE	TM	TE	TM	TE	TM	TE	TM
^40^	9.26	Yes	$95^{\circ }$	$95^{\circ }$	$96^{\circ }$	$95^{\circ }$	$99^{\circ }$	$98^{\circ }$	$98^{\circ }$	$98^{\circ }$	N/A	N/A	0.1	0.1
^41^	10.44	Yes	$90^{\circ }$	$90^{\circ }$	$92^{\circ }$	$92^{\circ }$	$98^{\circ }$	$98^{\circ }$	$99^{\circ }$	$99^{\circ }$	N/A	N/A	0.3	0.3
^43^	10.28	Yes	$97^{\circ }$	$97^{\circ }$	$98^{\circ }$	$99^{\circ }$	$92^{\circ }$	$99^{\circ }$	$71^{\circ }$	$94^{\circ }$	N/A	N/A	No	0.1
^47^	11.3	Yes	$99^{\circ }$	$99^{\circ }$	$99^{\circ }$	$99^{\circ }$	$98^{\circ }$	$98^{\circ }$	$86^{\circ }$	$86^{\circ }$	N/A	N/A	0.05	0.05
^53^	10.25	Yes	$92^{\circ }$	$92^{\circ }$	N/A	N/A	$99^{\circ }$	$99^{\circ }$	$87^{\circ }$	$96^{\circ }$	N/A	N/A	0.2	0.1
Proposed design	5.17	Yes	$99^{\circ }$	$99^{\circ }$	$99^{\circ }$	$99^{\circ }$	$99^{\circ }$	$98^{\circ }$	$93^{\circ }$	$98^{\circ }$	$70^{\circ }$	$93^{\circ }$	No	0.02

## Experimental results

**Figure 8:**
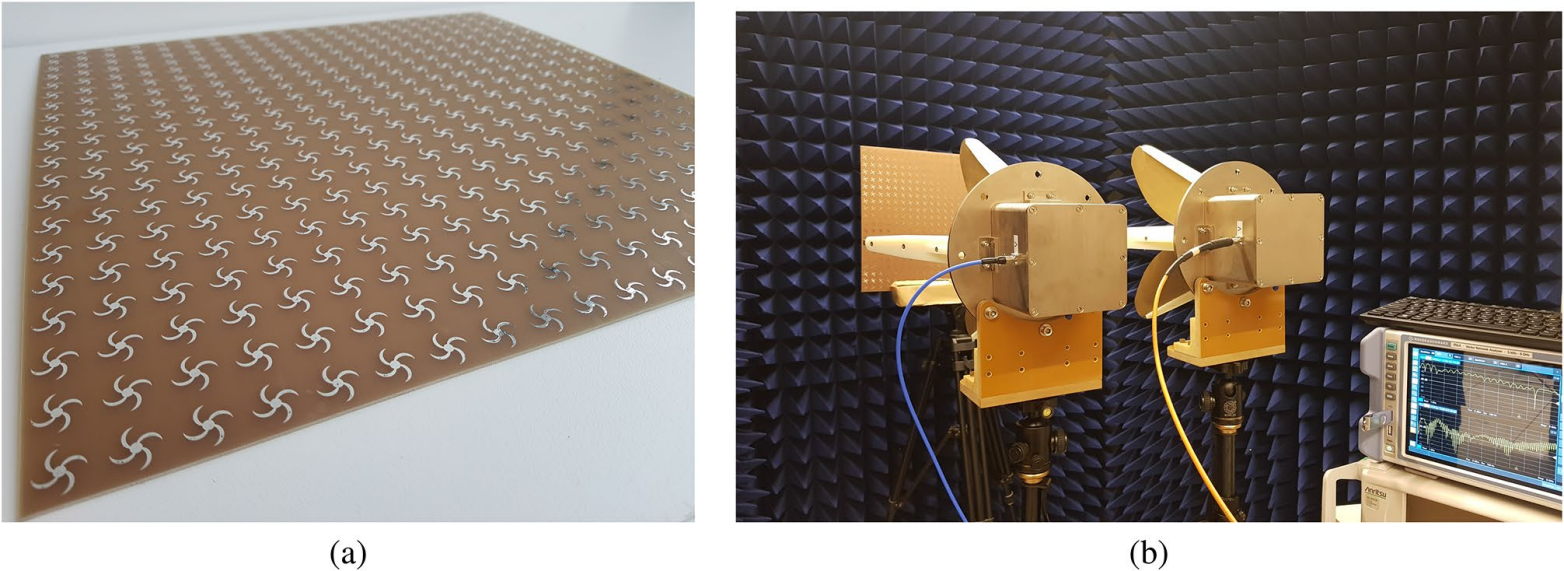
(**a**) Fabricated MPA, (**b**) measurement setup.

Figure 8a shows the proposed crescent shape MPA with four blades fabricated in 20 × 20 array using FR-4 substrate with $\varepsilon _r=4.4$, $tan\delta =0.02$ and thickness of $h=1.6$ mm, while the overall size is 360 mm × 360 mm. The ground plate and resonators are made from copper with thickness of $h=0.036$ mm and conductivity $\sigma =5.8 \times 10^7$ s/m. To measure $S_{11}$, $S_{21}$, and absorption ratio under normal incident wave angle, reference horn antennas are located one meter away from MPA to satisfy the far-field distance (more than10 $\lambda$). By rotating the reference antenna around the horizontal axis, absorption for different polarization angles are measured. However, to change the incident angle, two similar horn antennas, one as a transmitter and one as a receiver are used. Figure 8b shows the provided measurement setup.

**Figure 9:**
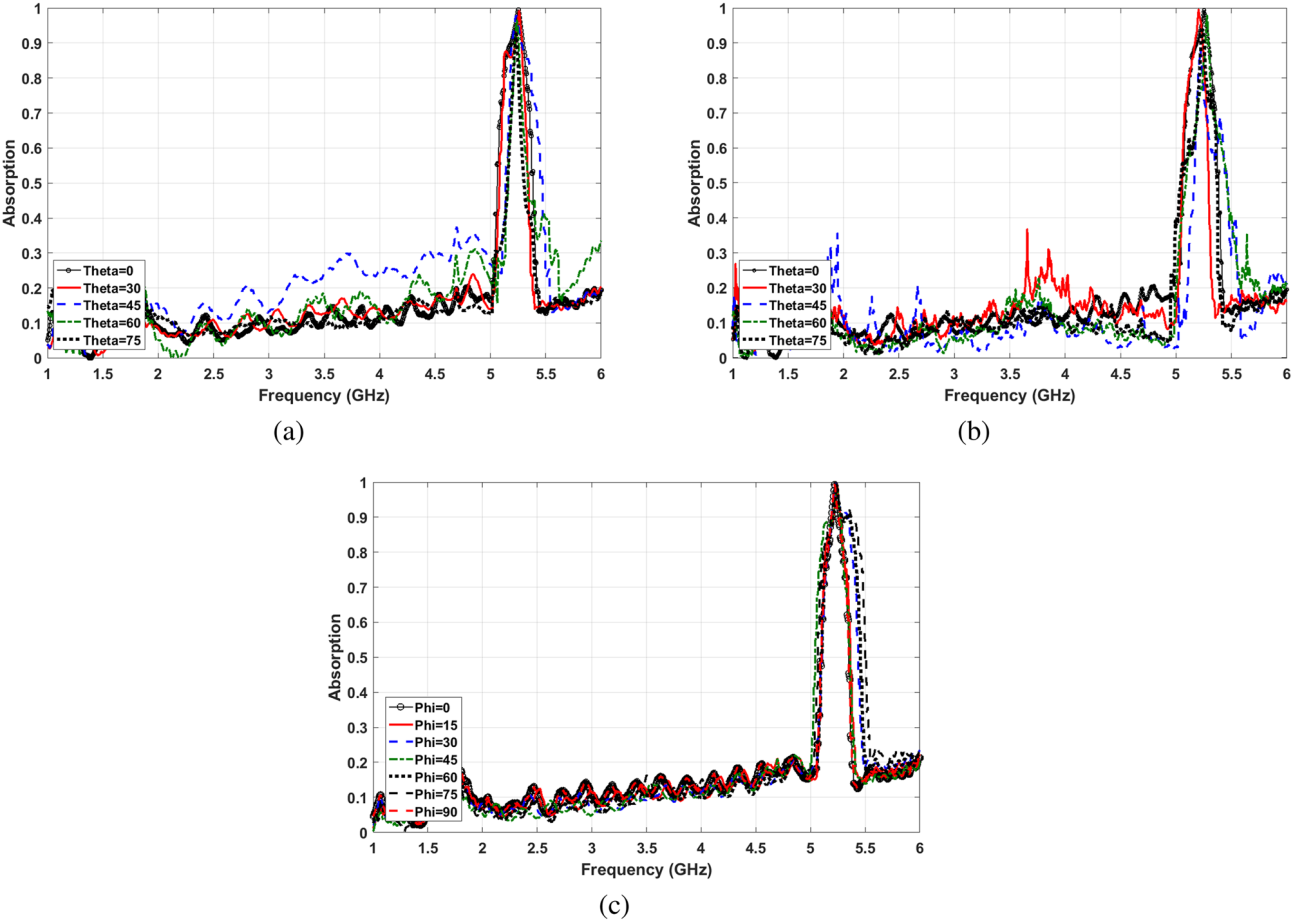
Angle insensitivity measurement results (**a**) different incident angles for TE waves, (**b**) different incident angles for TM waves, (**c**) different polarization angles.

Figure 9a,b show the measured absorption at different incident angles from $\theta =0^{\circ }$ to $\theta =75^{\circ }$ under TE and TM polarized radiated electromagnetic waves, respectively. These graphs imply that the absorption ratio of the structure remains higher than $95\%$ in all measured cases. Moreover, the peak frequency stays almost steady by increasing the wave incident angles. Similar behavior of the structure under TE and TM polarized incident waves at different angles, which follows simulation results, demonstrates the wide-angle insensitivity of proposed MPA. Moreover, Fig. 9c shows measurement results of the absorption ratio at different polarization angles. Almost unchanged absorption ratios in this graph confirm the polarization angle insensitivity of the proposed MPA structure due to the symmetric resonator shape.

## Discussion on adjusting sensitivity

Being insensitive for different incident angles has been investigated against TE and TM polarized waves in several works. Due to the difference in electric and magnetic responses of the structure facing TE and TM polarized waves, MPAs mostly do not show similar insensitivity for different modes. Based on Maxwell equations, the curl of the magnetic part and divergence of the electrical part of the incident waves lead to surface current and electrical load on the resonator, respectively. It is noteworthy that the electric charge creates a parallel current in the dielectric.

The perpendicular component of radiated waves to the structure plays a crucial role in electrical and magnetic responses. For TE polarized waves, there is no electric field in the propagation direction. Hence, the electrical response and created surface current are almost unchanged for different incident angles due to the smallest changes in the electric field. The situation is contrariwise for magnetic response and parallel current between the metal resonator and metal film. By increasing the incident angle, the perpendicular component of the magnetic part of the radiated waves is decreased. Then, the weakening of magnetic flux leads to impedance mismatching between air and structure, so the majority of EM waves are reflected.

On the other hand, for TM polarized waves, lack of the perpendicular element of the magnetic field causes stable parallel current. It is due to the almost unchanged magnetic part of radiated waves. However, the radiated power of the electric field decreases significantly for the higher angle of incident waves. Due to the shape of resonators, the MPAs show different absorption properties for TE and TM mode waves. They can be more insensitive against changing the incident angle for TE or TM waves. However, insensitivity against specific polarization has to be considered from the initial step of the design procedure.

The reported structures in literature are not able to adjust the sensitivity against TE and TM polarized waves. For instance, the circular shape resonator with four sectors, which has been investigated in^41^, shows better insensitivity for TE polarized wave for different circle sector angles ($\alpha$). This parameter specifies the width of each blade of the resonator. It should be noted that this structure has been reported in several papers^41,54^. This structure has been simulated to clarify the disability of a similar structure to adjust the insensitivity facing TE and TM modes.

The surface current distribution could justify a stable trend in better insensitivity for TE mode. As it is mentioned before, the magnetic field and surface current need to be unchanged to be insensitive for TE polarized waves. In the reported structure, the parts of the resonator with higher surface current density are almost fixed for different $\alpha$. Hence, the structure insensitivity for TE polarized waves remains acceptable by changing $\alpha$.

**Figure 10:**
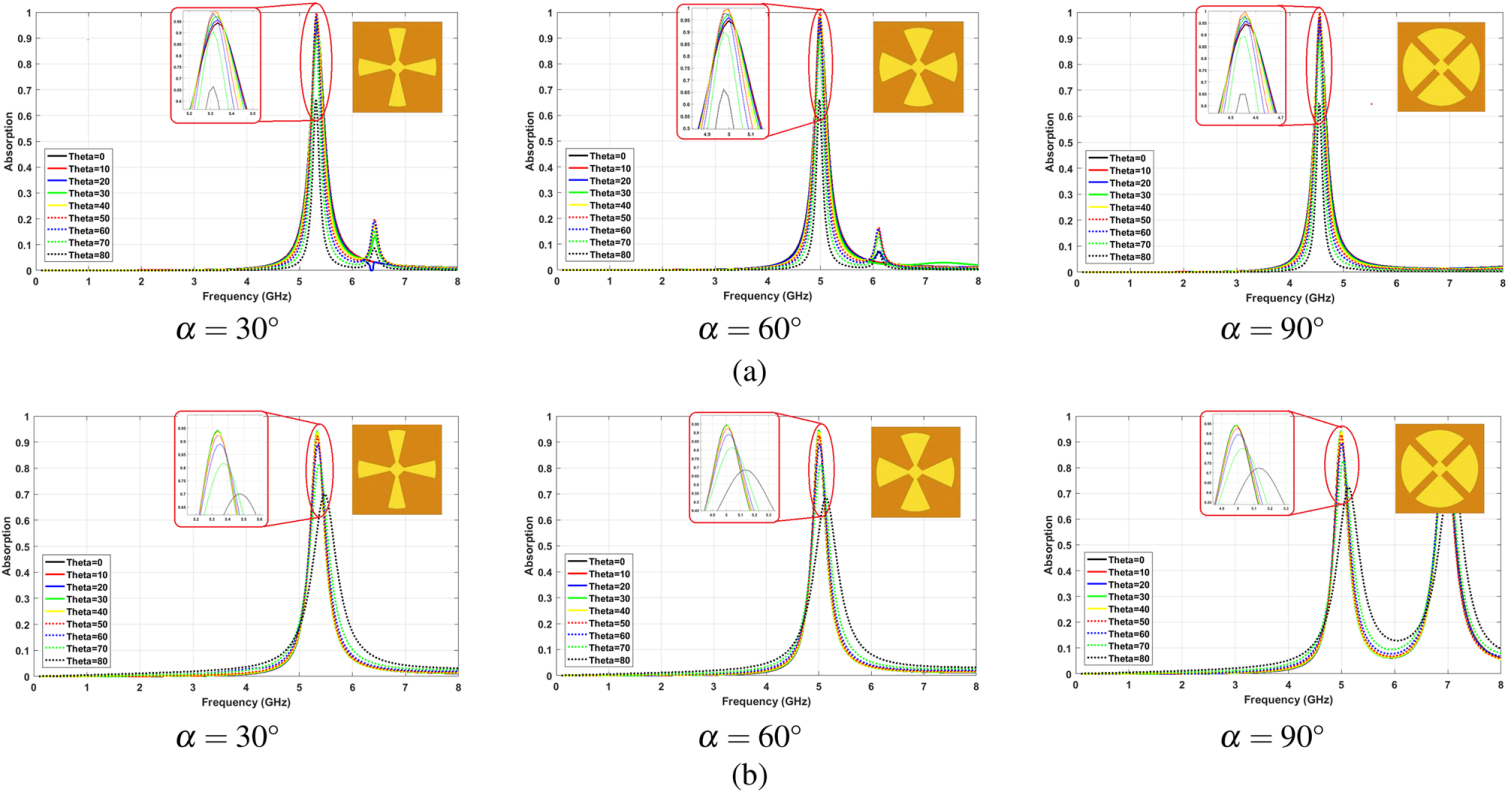
Absorption sensitivity trend by changing $\alpha$, (**a**) TE polarized waves, (**b**) TM polarized waves.

Figure 10a,b show the absorption characteristics for different incident angles by changing $\alpha$ for TE and TM polarized waves, respectively. As shown in Fig. 10, for various values of $\alpha$, the absorption efficiencies of the structures at a certain incident angle ($\theta$) are different. However, the sensitivity trend is constant for different values of parameters, and in all cases, the TE mode insensitivity is better than TM mode.

The proposed metamaterial absorber in this work shows a better absorption ratio and insensitivity for different incident angel up to $\theta =80^{\circ }$ compared to previously reported MPA. Furthermore, the particular semi-symmetric structure leads to adjustable absorption insensitivity for various incident angel against TE and TM polarized waves. Meanwhile, the absorption behaviour remains insensitive for different polarization angles. Thus, this feature can be applied in applications that require distinguishing between two modes. For instance, in radio transmission in the tunnel environment^55^, the tunnel has been assumed as a waveguide. However, the radiated waves cannot be categorised as pure TE or TM mode due to non-perfect conductive walls of the tunnel. In this case, the waves are classified as hybrid modes. To minimize the undesired mode in the transmission environment, the proposed MPA in this paper is a promising solution. It should be noted that the absorption insensitivity of proposed structure facing TE or TM polarized waves has to be considered based on application requirements prior to fabrication. By applying a slight change in the crescent blades width the aimed insensitivity is achievable.

**Figure 11:**
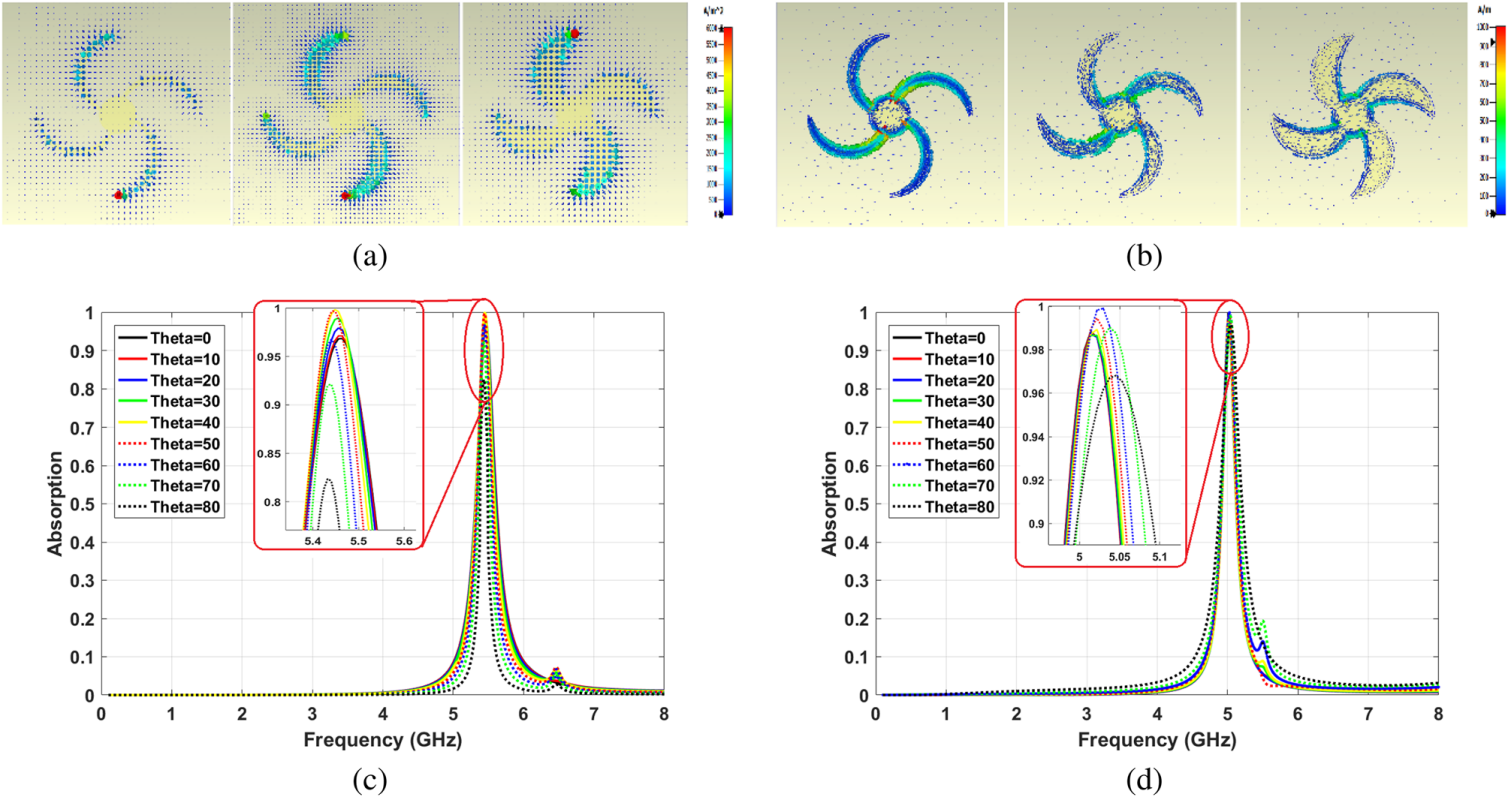
(**a**) Parallel current in structure with increasing Crescent width, (**b**) surface current in structure with increasing Crescent width, (**c**) TE absorption sensitivity for $w=2$ mm, (**d**) TM absorption sensitivity for $w=0.5$ mm.

In more detail, the surface current needs to be kept steady for various incident angles to have better insensitivity for TE mode. On the other hand, enhancing parallel current for higher incident angles leads to better insensitivity for TM polarized waves. Both these changes are achievable by tuning crescent shape blades parameters. As shown in Fig. 11a, by widening the blades, the parallel current is almost unchanged between the edge and metal film for TE mode waves under $\theta =80^{\circ }$. This is due to the minimal structural change in this area. However, area extension and decreasing the impedance of the microstrip line improve the surface current on the resonator. This phenomenon results in a more stable absorption ability in the wider blade $(w=2$ mm) for TE waves. Figure 11c shows that the absorption ratios at $\theta =70^{\circ }$ and $\theta =80^{\circ }$ reach to $92\%$ and $83\%$, which means $6\%$ and 13% improvement compare to the proposed blade $(w=1$ mm).

For TM mode waves, magnetic response and parallel current are almost fixed, and impedance mismatching can happen due to the weakening electrical response. Hence, the surface current should be improved to increase incident angle insensitivity. As can be seen in Fig. 11b, the surface current increases on narrower blades structure. Fig. 11d illustrates the absorption ratio for $w=0.5$ mm at $\theta =70^{\circ }$ and $\theta =80^{\circ }$ which are $99\%$ and $97\%,$ respectively. Compared to the purposed structure, $4\%$ and $5\%$ improvement can be seen.

## Conclusion

In this work, a semi-symmetric crescent shape of MPA has been investigated. Due to the unique geometry of the resonator, it shows remarkable insensitivity against the changing polarization and incident angles for both TE and TM mode. Based on simulation results, changing the polarization angels from $\phi =0^{\circ }$ to $\phi =90^{\circ }$ has a negligible effect on absorbing efficiency, which is $99\%$. In addition, the absorption ratio has been decreased from 99% to $70\%$ for TE mode waves when incident angles increased from $\theta = 0^{\circ }$ to $\theta = 80^{\circ }$. On the other hand, the absorption ratio has been changed from 99% to $93\%$ in the same range of incident angles facing TM polarized waves.

In Table 1, the proposed structure has been compared with some other polarization and incident angles insensitive structures in different aspects to show the sensitivity enhancement. Based on this comparison, in addition to the absorption ratio, the absorption frequency is more stable than previous reports.

Moreover, changing the width of crescent shape blades leads to manipulating the surface and parallel current in the structure. Therefore, unlike the reported structures in literature, the absorption characteristic can be adjusted to have better insensitivity for TE or TM modes based on application requisites. This feature can be applied to minimize the undesired mode in the transmission environment. In proposed MPA, the absorptivity for TE polarized waves at $\theta =80^{\circ }$ has been improved to $83\%$ by widening the crescent shape blades from $w=1$ mm to $w=2$ mm. Also, TM polarized waves absorption ratio has been increased up to $97\%$ when $w=0.5$ mm.

The optimum design to satisfy both TE and TM insensitivity has been fabricated, which consists of $20 \times 20$ unit cells. The similarity of measurement and simulation results confirm the accuracy of the design process.

## Methods

All the proposed metamaterial absorber design procedure has been done in commercial software CST STUDIO SUITE 2016 by using frequency-domain solver. The periodic boundary is applied for the unit cell, and the parametric studies have been performed under the normal incident angle. Floqute port has been used to investigate the sensitivity of structure against different polarization and incident angles.

## Measurement

The absorptivity has been extracted from the S-parameters that were measured by using the described setup in Fig. 9b. A two ports ROHDE & SCHWARZ ZNL6 vector network analyzer (VNA) and two AINFOMW quad-ridged horn reference antennas have been used to perform measurements. One of the antennas has been connected to port one, which is responsible for transmitting the signal with a specified power of − 5 dBm in our case. The other horn antenna, which is connected to port two of VNA, worked as a receiver for reflected signals from the MPA surface. The experiments have been done in the environment with minimum wave reflecting and scattering due to surrounding by wedge-trapped absorbing material in all directions. Prior to measuring S-parameter, the setup has been calibrated using a metal plate with the same size of MPA. These results were considered as base values to compare with S-parameter values when fabricated absorber was measured. In order to measure absorptivity at different incident angles, two antennas were moved equally around a circle with a diameter of 1 m. The $\theta$ is the angle between the perpendicular axis to the center of MPA and horn antenna in the azimuth plane. In the sequence of measuring absorptivity at different polarization angles, one horn antenna was placed on MPA perpendicular axis. Then $S_{11}$ was measured by rotating the horn antenna around the perpendicular axis. It is noteworthy that keeping both of the transmitter and receiver antennas in the same polarization angle leads to TE wave measurement. On the other hand, if two horns are positioned with the 90° difference between the polarization angle, the TM wave measurement setup is achieved.
